# Ethnic differences in calcium, phosphate and bone metabolism

**DOI:** 10.1017/S0029665114000068

**Published:** 2014-03-12

**Authors:** J. Redmond, L. M. A. Jarjou, B. Zhou, A. Prentice, I. Schoenmakers

**Affiliations:** 1 Elsie Widdowson Laboratory, Medical Research Council Human Nutrition Research, Cambridge CB1 9NL, UK; 2 Medical Research Council Keneba, The Gambia; 3 Department of Public health, Shenyang Medical College, 146 Huanghe North Street, Shenyang 110034, People's Republic of China

**Keywords:** Dietary intake, Diurnal rhythm, Calcium, Phosphate, Bone, Parathyroid hormone

## Abstract

The prevalence of osteoporosis and the incidence of age-related fragility fracture vary by ethnicity. There is greater than 10-fold variation in fracture probabilities between countries across the world. Mineral and bone metabolism are intimately interlinked, and both are known to exhibit patterns of daily variation, known as the diurnal rhythm (DR). Ethnic differences are described for Ca and P metabolism. The importance of these differences is described in detail between select ethnic groups, within the USA between African-Americans and White-Americans, between the Gambia and the UK and between China and the UK. Dietary Ca intake is higher in White-Americans compared with African-Americans, and is higher in White-British compared with Gambian and Chinese adults. Differences are observed also for plasma 25-hydroxy vitamin D, related to lifestyle differences, skin pigmentation and skin exposure to UVB-containing sunshine. Higher plasma 1,25-dihydroxy vitamin D and parathyroid hormone are observed in African-American compared with White-American adults. Plasma parathyroid hormone is also higher in Gambian adults and, in winter, in Chinese compared with White-British adults. There may be ethnic differences in the bone resorptive effects of parathyroid hormone, with a relative skeletal resistance to parathyroid hormone observed in some, but not all ethnic groups. Renal mineral excretion is also influenced by ethnicity; urinary Ca (uCa) and urinary P (uP) excretions are lower in African-Americans compared with White-Americans, and in Gambians compared with their White-British counterparts. Little is known about ethnic differences in the DR of Ca and P metabolism, but differences may be expected due to known differences in lifestyle factors, such as dietary intake and sleep/wake pattern. The ethnic-specific DR of Ca and P metabolism may influence the net balance of Ca and P conservation and bone remodelling. These ethnic differences in Ca, P and the bone metabolism may be important factors in the variation in skeletal health.

Abbreviations:
DR: diurnal rhythm
FGF-23: fibroblast growth factor-23
PTH: parathyroid hormone
uCa/uCr: urinary Ca/urinary creatinine
uP: urinary P

Ethnicity is generally defined as ‘the fact or sense of belonging to a particular ethnic group’^(^^1^^)^. An ethnic group shares a distinctive cultural and historical tradition, often associated with nationality, or region by which the group identifies itself and others recognise it^(^^1^^)^. This broad concept of ethnicity, encapsulating culture, lifestyle, identification, environment, biology and genetics, is what we refer to in this review.

## Ethnic differences in bone health

The prevalence of osteoporosis and the incidence of age-related fragility fracture vary by ethnicity. Kanis *et al*. report a greater than 10-fold variation in fracture probability between countries across the world^(^^2^^)^, and substantial differences are also seen between ethnic groups within a country. The age-adjusted incidence rates of hip and distal-forearm fractures in elderly West African men and women are shown to be substantially lower than those found in Britain^(^^3^^)^. Similarly, Yan *et al*. have reported a low incidence of hip fracture in Northern China, and the overall male-to-female ratio for hip fracture incidence was 1·15:1 in contrast with that of 0·34:1–0·43:1 in populations of most Western countries^(^^4^^)^. Differences are also found within the USA between ethnic groups. Age-adjusted hip fracture rates were reported to be 57/100 000 in African-American females, compared with 140/100 000 in White-American females^(^^5^^)^, with similar trends for men^(^^6^^)^. Also, Asian and Hispanic men and women living in the USA have lower hip fracture rates than their White-American counterparts^(^^5^^,^^7^^,^^8^^)^. It appears that across the world, White populations are consistently reported to be at a higher risk of fracture than other ethnic groups^(^^2^^–^^4^^,^^7^^,^^8^^)^. These differences cannot be fully explained by a lower bone mass in White populations. While African-Americans tend to have a higher bone mineral density than White-Americans, this is not the case for Asian-Americans, Asians or Africans. Fracture risk is multi-factorial and only partly depends on bone mass. Other factors influencing fracture risk are likely to be involved in the observed ethnic differences in bone health.

The multitude of factors determining fracture risk can be subdivided into extra-skeletal and skeletal factors. The former include age, gender, neuromuscular coordination, falls, and in the case of a fall, adiposity and soft tissue cushioning may absorb the force and thus influence the impact on the bone. Skeletal factors include bone mass, geometry, the rate of bone turnover and mineral metabolism. Mineral and bone metabolisms are intimately interlinked and this review focuses on ethnic variation in the metabolism of the bone forming minerals, Ca and phosphate (P).

### Ca and P metabolism

Bone consists of an extracellular mineralised matrix consisting of mineral salts (mainly hydroxyapatite (Ca_10_(PO_4_)_6_(OH)_2_) crystals) bound to proteins. The metabolic function of bone is crucial for Ca and P homoeostasis. Ca, P and bone metabolism are regulated by common factors, mainly parathyroid hormone (PTH), 1,25-dihydroxy vitamin D (1,25(OH)_2_D) and fibroblast growth factor-23 (FGF-23). The parathyroid gland detects the fluctuations in plasma ionised Ca (Ca^2+^) concentration through the Ca^2+^-sensing receptor. In response to a reduction in plasma Ca^2+^, PTH is secreted by the parathyroid cells. In the kidney, PTH binds to the PTH receptor and stimulates renal Ca reabsorption from the urinary filtrate^(^^9^^)^. This occurs through a multitude of actions, including the increase of the renal Na/K/Cl_2_ co-transporter to stimulate paracellular Ca reabsorption, and through the transient receptor potential channel TRPV5, to stimulate luminal Ca transfer^(^^9^^,^^10^^)^. PTH also stimulates the renal conversion of 25-hydroxy vitamin D (25(OH)D) to 1,25(OH)_2_D via the up-regulation of the enzyme 25-hydroxyvitamin-D-1-α-hydroxylase. This, in turn, increases the intestinal absorption of Ca and P. Both 1,25(OH)_2_D and PTH act together to stimulate osteoblasts to produce factors that activate osteoclastic bone resorption^(^^9^^)^, liberating Ca and P into the extracellular fluid^(^^9^^)^. At the same time, factors reflecting cellular function of bone are released into plasma, known as bone turnover markers.

FGF-23 is primarily a P-regulating hormone and increases in response to a high plasma P and 1,25(OH)_2_D^(^^11^^)^. In normal physiology, FGF-23 is mainly produced by the osteocytes, but the FGF-23 mRNA is also expressed in the osteoblasts and the bone lining cells^(^^12^^)^. FGF-23 acts on the kidney to increase renal P excretion via down-regulation of the renal type-II transporters, NaP-IIa and NaP-IIc in the proximal tubule. In addition, FGF-23 acts to decrease the plasma 1,25(OH)_2_D^(^^11^^)^. The net effect is to decrease plasma P concentration. This partly counteracts the potential PTH- and 1,25(OH)_2_D-induced release of P from the skeleton and the up-regulation of P absorption in the intestine.

Although an elevated plasma PTH increases bone turnover, the net effect on bone depends on the pattern and the duration of exposure to a high plasma PTH. A sustained high plasma PTH concentration due to primary or secondary hyperparathyroidism can lead to bone loss, whereas anabolic effects occur with intermittent exposures^(^^13^^–^^16^^)^. Therefore, PTH may have contrasting effects on the bone depending upon the length and degree of exposure. The action of PTH may differ between different ethnic groups^(^^17^^)^.

### Modulators of Ca and P metabolism and their regulators

Hormonal regulation of mineral metabolism ensures tight control of plasma Ca and P concentrations over a wide range of nutrient intakes and absorption. The plasma concentrations of the calciotropic hormones are therefore influenced by dietary factors. Many other factors influence their concentration. Kidney function influences plasma PTH, FGF-23, 1,25(OH)_2_D and 25(OH)D concentrations^(^^18^^)^. Plasma PTH is also influenced by age, BMI and fasting^(^^19^^)^. Plasma 25(OH)D is further influenced by levels of skin exposure to UVB-containing sunshine (related to clothing, sunscreen, air pollution) and skin tone^(^^20^^,^^21^^)^. Some of these modulators are known to differ by ethnicity, reflecting differences in lifestyle, physiological or genetic factors. Moreover, Ca and P metabolism exhibits variation during the day and the night in healthy individuals. This is known as the diurnal rhythm (DR). Data comparing the DR between ethnic groups are limited. They may be expected to differ, as they are influenced by lifestyle factors, and will be summarised later in this review.

### Diurnal rhythms of Ca and P and regulators

DR refer to patterns of physiology, activity or behaviour that follow the day–night cycles, and these may be controlled by an underlying endogenous biological mechanism, known as the circadian rhythm^(^^22^^)^. DR repeat every 24 h and are associated with external factors such as sleep–wake cycles, light–dark cycles, meals or posture.

**Fig. 1.**
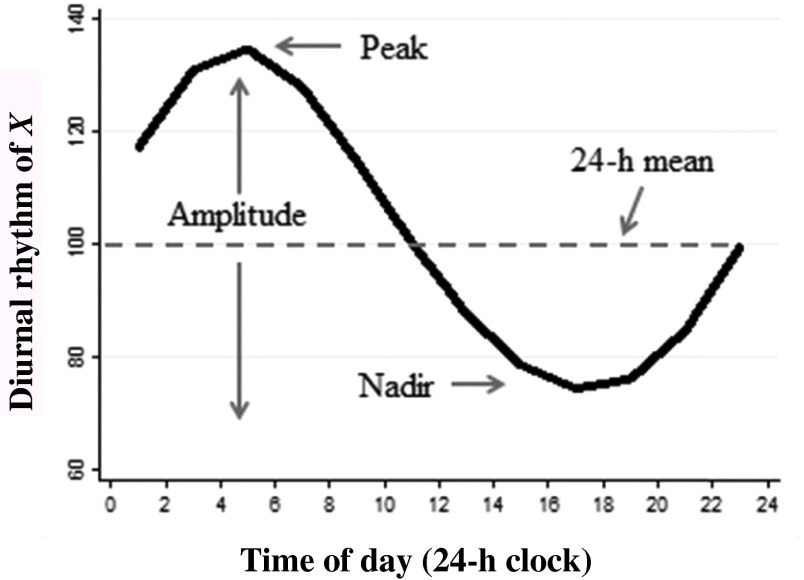
Schematic representation of the diurnal rhythm of biological data.

Biological data that follow a DR usually exhibit a change over time in a smooth and continuous manner, ascending to a maximum value, the peak and decreasing to a minimum value, or nadir. The amplitude refers to the variation over the day. The average value of a measurement can be derived from the diurnal curve and is referred to as the 24-h mean, as shown in Fig. 1.

The DR of Ca, P and bone metabolism is known to be at least partially dictated by exogenous factors, such as dietary and sleep–wake patterns. The majority of this research has been conducted in White and Western populations.

#### The diurnal rhythms of Ca and P metabolism

**Fig. 2.**
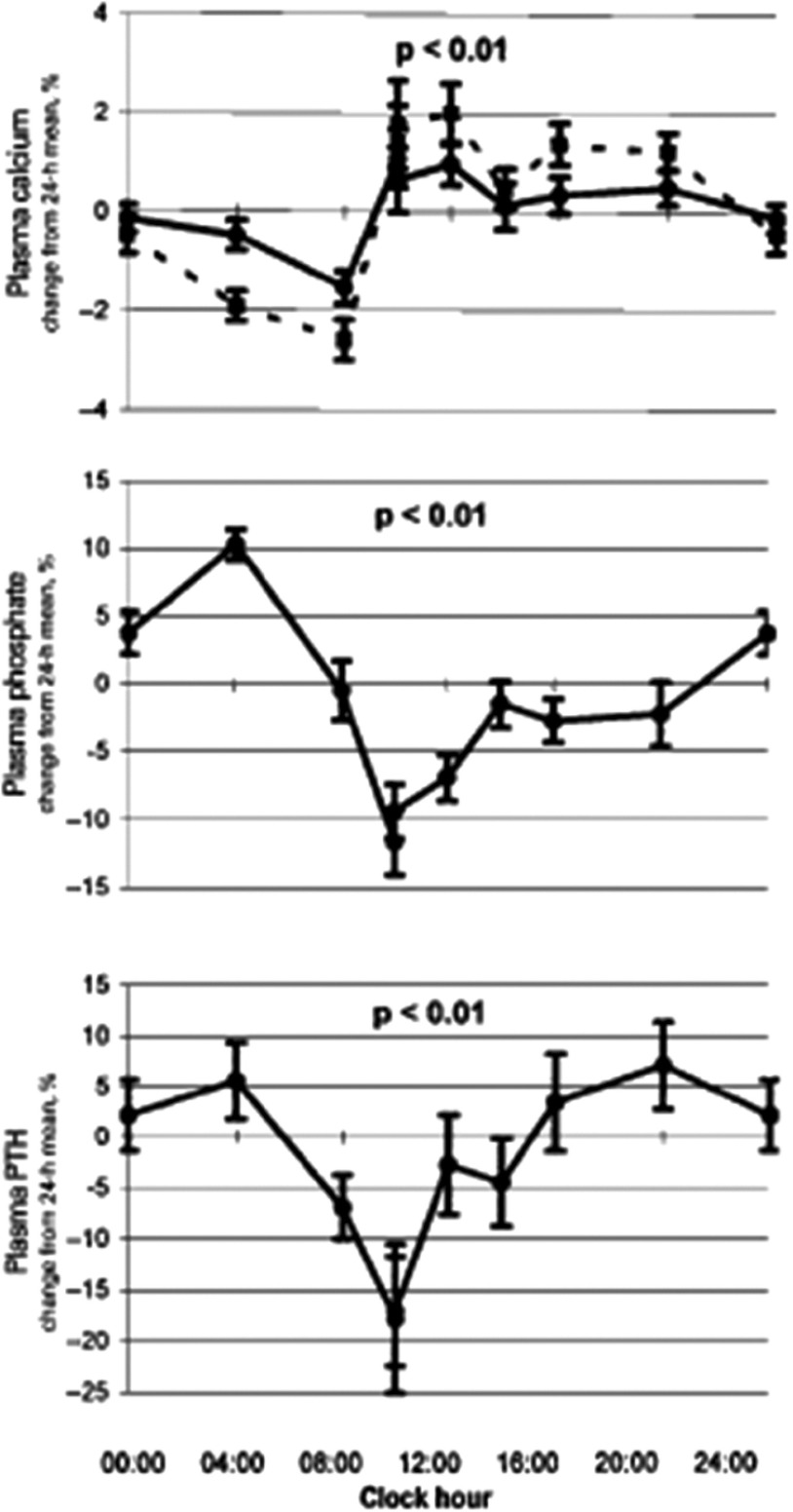
Diurnal variation in total plasma Ca (albumin-adjusted: bold lines and unadjusted: broken line), phosphate (P) and parathyroid hormone (PTH) (change from 24 h mean, % mean and sem) in healthy post-menopausal women. *P*-value indicates the significance of the diurnal variation. Reproduced from^(^^25^^)^ with permission. Figure 2 was granted permission for reproduction by Copyright Clearance Center's RightsLink service by the European Journal of Endocrinology. License no.: 3213570527941.

Plasma total Ca is reported to have a nadir at night and a day-time peak in men and women (see Fig. 2)^(^^23^^,^^24^^)^. It remains unclear whether this is related to the DR of plasma albumin^(^^24^^,^^25^^)^. Urinary Ca (as expressed in mmol/h or as a ratio to urinary creatinine (uCa/uCr)) follows a significant DR with a night-time nadir and a day-time peak in both younger and older adults^(^^24^^,^^26^^,^^27^^)^. Plasma P tends to follow a DR with an increase during the day, with a peak late in the evening and night and a nadir in the early morning, both in post-menopausal women^(^^24^^)^ and healthy adult men and women^(^^28^^)^. Urinary P excretion (as measured by mmol/h or as uP/uCr), exhibits a nocturnal nadir and a day-time peak^(^^24^^,^^26^^)^; this is consistent with the DR described for uCa excretion. The nocturnal decrease in renal Ca and P excretion is likely to partly reflect the biological response to nocturnal fasting by conserving minerals at the kidney level. The nocturnal decrease in urinary mineral excretion may also be associated with the DR in plasma PTH.

#### The diurnal rhythm of parathyroid hormone

Plasma PTH concentration exhibits a well-documented DR with two peaks in healthy people, one in the afternoon/evening, and one at night (see Fig. 2)^(^^23^^,^^24^^,^^26^^,^^29^^)^. The timing of the nocturnal peak of plasma PTH concentration corresponds to a decrease in uCa/uCr excretion and (with a time lag) to the decrease in uP/uCr^(^^26^^)^. This is suggestive of a physiological role for the DR of PTH in regulating renal Ca and P excretion^(^^26^^)^.

Studies of the pathological conditions have shown changes in the DR of PTH^(^^25^^,^^30^^–^^32^^)^. A comparative study between osteoporotic and healthy women reported a significant DR of PTH in both the groups, but with differences observed between the groups^(^^33^^)^. In osteoporotic women, there was a higher 24-h mean of plasma PTH concentration with a smaller nocturnal increase, and the nocturnal peak occurred at an earlier time compared with healthy women^(^^33^^)^. These findings suggest that the DR of plasma PTH may be important for overall mineral and bone metabolisms. The physiology of the DR of plasma PTH has been reviewed elsewhere in detail^(^^32^^)^.

#### The diurnal rhythms of 1,25(OH)_2_D, vitamin D binding protein and fibroblast growth factor-23

Plasma 1,25(OH)_2_D and vitamin-D-binding-protein were found to have a significant DR in postmenopausal women, although the free fraction of 1,25(OH) _2_D (i.e. not bound to the vitamin-D-binding-protein) did not vary over the 24-h cycle^(^^24^^)^. Only one study is available which investigates the DR of FGF-23 and has reported that FGF-23 did not have any significant DR in healthy children and adults and patients with X-linked hypophosphataemia, although a significant DR was observed for its biological co-factor, Klotho^(^^34^^)^.

#### The diurnal rhythms of bone turnover markers

Biochemical markers of bone turnover exhibit a DR, with a night-time peak and a day-time nadir^(^^23^^,^^35^^–^^37^^)^. In a study of healthy men, the bone resorption marker, carboxy-terminal-collagen-crosslinks had a night-time peak that was 66 % greater than the 24-h mean^(^^37^^)^. The DR of the bone formation markers osteocalcin and the bone-alkaline-phosphatase exhibits less variation than the DR of bone resorption, and the amplitude is typically only 10–20 % of the 24-h mean^(^^23^^,^^38^^)^; which may relate to the differences in metabolism or excretion of the markers^(^^35^^)^.

The DR of the bone turnover markers is partially dictated by exogenous factors; it is altered by fasting and food intake^(^^35^^,^^39^^,^^40^^)^. It was reported that plasma concentrations of the bone resorption marker carboxy-terminal-collagen-crosslinks, and to a lesser extent the bone formation markers procollagen-type-I-N-terminal-propeptide and osteocalcin, were significantly higher after 9 h fasting compared with the fed state^(^^35^^)^. There are acute post-prandial changes in bone turnover that may be associated with changes in the activity of mature bone cells^(^^40^^)^. Potential mediators of this response include calciotropic hormones, growth hormone, pancreatic peptides and incretin hormones. The DR of bone resorption has also been described to be modulated by the pattern of dietary Ca intake^(^^41^^–^^43^^)^. Further information may be found in a recent review^(^^40^^)^.

Ahmad *et al*. reported an association between the DR of plasma concentrations of PTH and bone turnover markers; the timing of the peak concentrations of the carboxy-terminal-collagen-crosslinks and the procollagen-type-I-N-terminal-propeptide lagged behind the peak concentration of PTH by about 3 h^(^^25^^)^. The results of this study suggest that PTH may have some regulatory role in the DR of bone resorption and formation, as reflected by the markers of the bone turnover^(^^25^^)^, although this has not been consistently seen^(^^29^^)^.

Taken together, these results suggest the homoeostasis of plasma Ca at night, in the absence of Ca intake, is maintained by an enhanced bone resorption and also by an enhanced renal reabsorption of Ca^(^^44^^)^. This homoeostasis is in part, but not completely, mediated via a nocturnal increase in plasma PTH. Differences in DR of Ca, P and bone metabolisms between healthy individuals and patients with osteoporosis are reported^(^^30^^,^^31^^)^, particularly in PTH and uCa excretions. This indicates that they may be important for Ca balance and skeletal integrity.

Little is known about the influence of ethnicity on these DR. Differences may be expected between ethnic groups due to differences in lifestyle factors, including the timing of sleep–wake patterns, the pattern of dietary intake and total dietary intake. Differences in dietary intake and other factors associated with Ca and P metabolisms may vary considerably between ethnic groups, and will be discussed in this review.

## Ethnic differences in Ca and P metabolism

### Ethnic differences in dietary intake

Differences in food availability, cultural preferences and socioeconomic factors contribute to differences in dietary intake between ethnic groups within and between countries.

Ca is present in a wide range of foods, although the bioavailability varies depending on the source. Milk and milk products have a high Ca content. Plant sources of Ca include pulses, whole-grains, nuts, some dried fruit, tofu and green vegetables. On the population level, Ca intakes can vary by approximately 5-fold depending on the types of foods consumed^(^^45^^)^. They range from very low intakes (200–400 mg/d) in some parts of Asia (e.g. China, Japan) and Africa (e.g. The Gambia, Kenya)^(^^46^^–^^51^^)^ with Ca from vegetables, legumes, cereals and nuts accounting for much of the intake^(^^46^^,^^51^^)^ to higher intakes in mostly White populations (>750 mg/d) of the USA, the UK and the Northern European countries^(^^52^^–^^54^^)^.

Protein-rich foods (milk, meat, poultry and fish) and cereal grains provide the majority of dietary phosphorus intake^(^^55^^)^. The Ca:P ratio may be important, but little is known about their ethnic differences in dietary Ca:P ratio. Spencer *et al*., showed that although Ca balance did not change with varying intakes of dietary P, the higher P intake resulted in a significant decrease of the daily uCa output^(^^56^^)^. This may be related to the lower availability of Ca for intestinal absorption due to its binding to P. The effect of the dietary Ca:P ratio on bone health is inconclusive.

Differences in dietary intake may exist between the ethnic groups living in the same geographical region, for example, within the USA, as detailed in the next section. Within the UK, 42 % of women of African or Caribbean origin, and 36 % of women of South-Asian or other Asian ethnic origin had Ca intakes below the lower reference nutrient intake, compared with only 8 % of White-British women^(^^52^^,^^57^^)^. Also, a Canadian study comparing Western-born Chinese, recent Chinese migrants to Canada and White-Canadians found differences in the macro- and the micro-nutrient intakes between these groups^(^^58^^)^.

In the remainder of this review, we will focus on selected ethnic groups, describing Ca and P intake and the influence this has on Ca and P regulation and bone metabolism. The focus will be to compare and contrast Ca and P metabolism between: (a) African-American and White-Americans in the USA, as an example of the differences between groups in the same geographical location; (b) African in the Gambia and White-British in the UK and (c) Chinese in Northern China and White-British in the UK, as examples of differences between the ethnic groups living in their environment of origin.

### Ethnic differences in Ca and P metabolism: African-Americans compared with White-Americans

#### Ethnic differences in dietary Ca and P intakes in the USA

Within the USA, ethnic groups differ in dietary Ca intake. White-Americans have a Ca intake close to the recommendations (∼900 mg/d), while African-Americans have a lower Ca intake at all stages of life, as documented in the National Health and Nutrition Examination Survey^(^^53^^)^ and other studies^(^^44^^,^^59^^)^. African-Americans are reported to consume the same or less dietary P than White-Americans^(^^60^^)^.

Some studies have reported greater fractional and total intestinal Ca absorption in African-Americans compared with White-American children and adolescents^(^^61^^,^^62^^)^. However, Bell *et al*. did not find any differences in fractional intestinal Ca absorption, despite the finding of lower serum 25(OH)D concentration and 24-h uCa excretion and higher serum 1,25(OH)_2_D in African-Americans than in White-American adolescents^(^^63^^)^.

#### Ethnic differences in 25(OH)D in the USA

After exposure to a single standard dose of UV radiation, there is less dermal synthesis of vitamin D_3_ from 7-dehydrocholesterol in darker skin because of the absorption of UVB-light by the skin pigment^(^^64^^)^, although the total capacity appears to be similar in African-Americans and White-American young adults when exposed to unlimited sunshine^(^^65^^)^. Consequently, when living in temperate climates, darker skinned individuals tend to have lower plasma 25(OH)D concentrations. This is reflected in data obtained within the USA, including the National Health and Nutrition Examination Survey, where for similar amounts of UVB-containing sunshine exposure, African-Americans have lower 25(OH)D in winter and summer^(^^59^^,^^66^^,^^67^^)^.

The lower plasma 25(OH)D concentration may also be partly explained by a higher BMI on average in African-Americans, as BMI is inversely associated with plasma 25(OH)D^(^^68^^)^. However, Coney *et al.* report that ethnic differences in plasma 25(OH)D remain after adjustment for body weight, percentage body fat and BMI^(^^69^^)^. The lower 25(OH)D may be possibly explained partly by genetic variations in vitamin D synthesis, the vitamin-D-binding-protein and/or metabolism between ethnic groups^(^^66^^,^^70^^)^.

#### Ethnic differences in regulatory hormones in the USA

Plasma PTH, 1,25(OH)_2_D and urinary cyclic-adenosine-3′,5′-monophosphate (an indicator of renal PTH activity) have been reported to be higher in African-Americans compared with White-American adults^(^^71^^–^^73^^)^. A lower vitamin D status, in addition to a lower Ca intake, may explain these findings^(^^72^^)^, since PTH is a primary regulator of Ca metabolism, as described earlier. The higher plasma PTH in African-Americans, however, may not lead to a similarly increased rate of bone turnover or risk of osteoporosis as it does for White-Americans. A comparative study of African-American and White-American women matched for age and weight, reported that, despite having lower plasma 25(OH)D and higher plasma PTH, African-American women have a higher bone mineral density and bone mineral content and lower bone turnover rate^(^^59^^)^. This was confirmed by other studies reporting lower concentrations of bone turnover markers in African-Americans despite a similar or higher plasma PTH as White-Americans^(^^72^^,^^74^^)^. This is further underpinned by the results of a controlled PTH infusion study showing a lower rise in the markers of bone resorption in African-American compared with White-American premenopausal women in response to the same amount of infused PTH^(^^17^^)^. Furthermore, histomorphometric analysis of biopsies of the iliac crest after double-tetracycline labelling showed that the bone formation rate in African-American adults was only one-third that of White men and women^(^^75^^)^. In addition, the relationship between plasma 25(OH)D concentration and bone outcomes differs by ethnicity; a lower plasma 25(OH)D is associated with a lower bone mineral density in White-Americans, but not African-Americans^(^^67^^)^. Taken together, these pieces of evidence suggest that the bone resorptive response to PTH differs between these groups, and it has been suggested that African-Americans have a relative skeletal resistance to PTH^(^^67^^)^.

#### Ethnic differences in renal mineral excretion in the USA

Studies comparing ethnic groups in the USA have shown that the African-American adults have lower daily uCa and uP excretions across all ages^(^^59^^,^^62^^,^^72^^,^^76^^)^. Postmenopausal African-American women excrete on average 65 mg less uCa/d and 120 mg less uP/d than White-American women^(^^77^^)^. In steady-state conditions of mineral ion balance, the daily urinary mineral excretion equates to net absorption from dietary sources^(^^76^^)^; accordingly, it has been speculated that the lower urinary mineral excretion among African-Americans is due to a lower intestinal absorption of Ca and P. This, however, needs to be interpreted with consideration of dietary intake. As mentioned earlier, when compared with White-Americans, African-Americans consume less dietary Ca and the same or less dietary P^(^^60^^)^. It has been demonstrated in adolescents and adult women that lower daily uCa excretion in African-Americans is primarily determined by a higher renal Ca conservation rather than lower fractional intestinal Ca absorption^(^^63^^,^^78^^)^. These differences cannot be fully explained by a higher plasma PTH and a 1,25(OH)_2_D concentration in African-Americans. A lower 24-h Ca excretion was reported in African-Americans compared with White-American adolescent girls across a range of controlled dietary Ca intakes, and at similar concentrations of plasma 25(OH)D, 1,25(OH)_2_D and PTH^(^^62^^)^. This was confirmed by a study by Gutierrez *et al.* examining ethnic differences in postprandial urinary fractional mineral excretion between African-American and White-American adults in samples collected for 4 h following a standardised meal^(^^76^^)^. Despite similar plasma PTH and FGF-23 concentrations, African-Americans had ∼35 % lower fractional Ca excretion and ∼30 % lower postprandial fractional P excretion than White-Americans^(^^76^^)^. These pieces of evidence suggest that African-Americans have a proportionally higher renal mineral conservation than White-Americans.

Overall, there appears to be strong evidence supporting the biologically relevant ethnic differences across all aspects of Ca and P metabolism and in the concentrations and responses to the regulators between African-Americans and White-Americans. The differences described in the skeletal responsivity to PTH, the rate of bone remodelling and the differences in Ca and P retentions may be associated with ethnic differences in bone mass and fracture risk (Table 1).

**Table 1.** Factors associated with ethnic differences in Ca, P and bone metabolism within the USA

Parameter	African-Americans *v.* White-Americans*
Osteoporosis/fracture risk^(^^5^^–^^7^^)^	↓
Dietary Ca^(^^44^^,^^53^^,^^59^^)^	↓
Dietary P^(^^60^^)^	↓/=
Plasma 25(OH)D^(^^59^^,^^66^^,^^67^^)^	↓
Plasma 1,25(OH)_2_D^(^^71^^–^^73^^)^	↑
Plasma PTH^(^^59^^,^^71^^–^^74^^)^	↑
Bone turnover markers^(^^59^^,^^74^^)^	↓
Renal Ca and P excretion^(^^59^^,^^72^^,^^77^^)^	↓

PTH, parathyroid hormone.

* Arrows indicate the differences compared with White-Americans.

### Ethnic differences in Ca and P metabolism: The Gambia *v.* the UK

#### Ethnic differences in dietary intake: The Gambia *v.* the UK

Dietary Ca intake in The Gambia, West Africa, is very low (200–400 mg/d)^(^^47^^,^^79^^,^^80^^)^. The main contributors to daily Ca intake are leaves, fish, locust beans, cereals, groundnuts and local salt, while milk is reported to account for only 5 % of Ca intake^(^^46^^)^. In the UK, milk and milk products contribute substantially to dietary Ca intake (approximately 56 %). Daily Ca intake is generally close to recommendations (760–960 mg/d) with some regional variations^(^^52^^)^. We have previously reported that the dietary Ca and Ca:P intakes were lower in rural Gambian compared with White-British adolescents and older adults from Cambridge^(^^47^^,^^80^^)^.

#### Ethnic differences in 25(OH)D: The Gambia *v.* the UK

Vitamin D status in The Gambia is generally higher than in the UK. Plasma 25(OH)D was reported to be 60–100 nmol/l in Gambian men and women throughout the year across all ages and in pregnancy^(^^21^^,^^80^^–^^83^^)^. In the UK, vitamin D status undergoes seasonal variation. The UK National Diet and Nutrition Survey reported mean annual 25(OH)D concentrations as 45·6 (sd 22·6)nmol/l for men and 49·6 (sd 25·6)nmol/l for women aged 19–64 years^(^^84^^)^.

#### Ethnic differences in regulatory factors: The Gambia *v.* the UK

Plasma PTH and 1,25(OH)_2_D concentrations are raised in Gambians compared with White-British subjects, and this is likely the result of their very low Ca intake^(^^80^^,^^82^^,^^85^^)^. A higher plasma PTH in the Gambia is found in conjunction with higher plasma concentrations of markers of osteoblast activity, with or without the concomitant higher markers of osteoclast activity^(^^47^^,^^82^^)^. Therefore, it appears that the higher plasma PTH in The Gambia is associated with greater bone turnover, which is similar to the findings reported in South Africans^(^^86^^)^, but is in contrast to the lower bone turnover and the reported skeletal resistance in African-Americans^(^^17^^)^. These findings are confirmed by a detailed study investigating the bone response after oral-P-induced PTH secretion^(^^47^^)^. This study showed no proportional differences between Gambians and White-British adults in the skeletal response to a raised plasma PTH^(^^47^^)^, suggesting no difference in their relative skeletal sensitivity to PTH.

In White-British and other White populations, a high plasma PTH has been shown to be related to a lower hip bone mineral content^(^^87^^)^ and pathologically elevated plasma PTH is a significant risk factor for osteoporosis and fracture^(^^88^^)^. In contrast, the chronically raised plasma PTH concentration that has been reported in the adults from The Gambia^(^^80^^,^^82^^,^^85^^)^, is not associated with a high incidence of fracture on a population level^(^^89^^)^. Schnitzler suggested that high bone turnover may remove fatigue damage and improve bone quality^(^^86^^)^ and is a plausible explanation for the very low fracture rates in West Africa.

#### Ethnic differences in renal mineral excretion: The Gambia *v.* the UK

There is evidence for differences in renal mineral conservation between The Gambia and the UK. Aspray *et al*. found that the 24-h uCa and uP outputs were both significantly lower in the rural Gambian adult women, particularly after menopause, compared with White-British women^(^^82^^)^. Further investigations of renal Ca handling and the influence of dietary intake in elderly men and women from the Gambia and the UK showed that the 2-h fasting uCa/uCr excretion was significantly lower in Gambian subjects. The UCa/uCr remained lower in Gambian subjects even after statistical adjustment for dietary Ca intake and for glomerular filtration rate, body weight, height and body surface area^(^^90^^)^. There were also differences in the fractional excretion of Ca (i.e. the percentage of Ca filtered by the kidney which is excreted in the urine), and in the tubular maximum of Ca (i.e. the maximum renal tubular mineral reabsorption, a threshold beyond which Ca is excreted in the urine)^(^^90^^)^. Similar trends were observed for uP and these differences also remained after statistical adjustment for dietary intake.

Taken together, these pieces of evidence suggest important differences in the Ca and P regulatory axes between White-British and Gambian adults, and between Gambians and African-Americans.

### Ethnic differences in Ca and P metabolism: China *v.* the UK

There are fewer data available on the differences between White-European and Chinese populations. The limited data available are summarised below.

#### Ethnic differences in dietary intake: China *v.* the UK

The traditional Chinese diet is low in Ca because of the low consumption of milk and milk products; Ca intake comes mainly from the consumption of green vegetables, legumes, cereals and small fish^(^^51^^,^^91^^)^. There is also considerable geographical dietary variation within China^(^^48^^)^. Hu *et al.* reported that the mean Ca intake ranged from 200 to 700 mg/d depending on the region^(^^48^^)^. Zhang *et al.* reported an increasing trend in the daily intake of Ca in Chinese adults between 1991 and 2009. Despite this, the average Ca intake in 2009 was 400 and 352 mg/d for men and women, respectively^(^^51^^)^. Other studies report Ca intakes were typically below 500 mg/d^(^^92^^,^^93^^)^. We have previously reported that older Chinese adults in Shenyang, Northern China have lower dietary Ca and P intakes and a lower dietary Ca:P ratio compared with their White-British counterparts^(^^47^^)^.

#### Ethnic differences in 25(OH)D: China *v.* the UK

Zhang *et al*. reviewed publications measuring the plasma 25(OH)D in China in various age groups and in different areas of China^(^^94^^)^. It was found that the vitamin D deficiency (defined as plasma 25(OH)D concentration <25 nmol/l) is observed in the Chinese population in almost all age groups and areas. For example, a high prevalence of deficiency was reported in Beijing (40°N) in the autumn and in Nanjing (latitude 31°N) during the winter months^(^^94^^)^. In women from Nanjing, the mean plasma 25(OH)D concentrations were 22 and 32 nmol/l in the winter and the summer, respectively^(^^95^^)^. In our studies, we have also reported significantly lower plasma 25(OH)D concentrations in the Chinese men in Shenyang (42°N) compared with their White-British counterparts in Cambridge (52°N) in late winter but not in summer^(^^87^^)^.

The aetiology of vitamin D deficiency in China is likely to be multifactorial. At latitudes >30°N, there is little to no cutaneous synthesis of vitamin D during the winter; the length of the ‘UVB winter’ depends on the latitude and is longer further in the North^(^^96^^)^. In addition, there may be avoidance of skin exposure to UVB from sunshine for cultural reasons, and high levels of air pollution in some areas^(^^94^^)^. Foods rich in vitamin D in the Chinese diet are scarce, and there is limited availability or consumption of vitamin D fortified products^(^^94^^)^.

#### Ethnic differences in the regulatory factors: China *v.* The UK

**Fig. 3.**
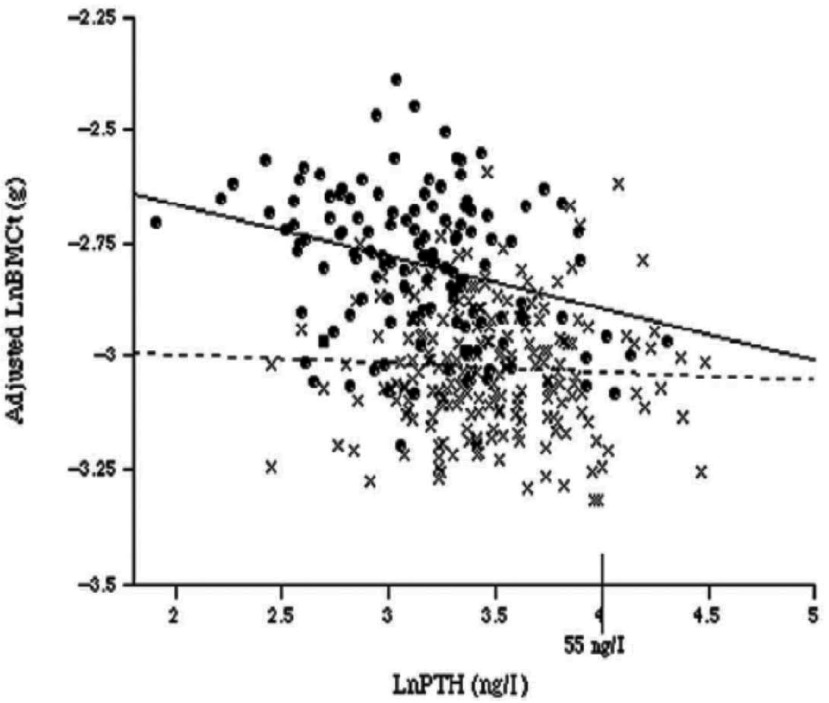
The relationship between Ln parathyroid hormone (PTH) and Ln bone mineral content (BMCt) at the femoral trochanter adjusting for bone area, weight, height, age and sex in Shenyang (x) and Cambridge (•) subjects. Reprinted from^(^^88^^)^ with permission from Elsevier. Figure 3 was granted permission for reproduction by Copyright Clearance Center's RightsLink service by Elsevier. License no.: 3196500798854.

We have previously reported that Chinese adults from Shenyang, Northern China had a higher plasma PTH concentration than White-British adults in winter months^(^^87^^)^, although no ethnic differences were reported in data from the summer and winter combined^(^^47^^)^. This finding is a likely result of lower Ca intake and, in winter, a lower plasma 25(OH)D in older Chinese adults than in White-British counterparts^(^^47^^,^^87^^)^. The relationship between plasma PTH and 25(OH)D differed between the two ethnic groups; Chinese adults had a higher plasma PTH for a given plasma 25(OH)D concentration^(^^87^^)^. In older White-British adults, a negative relationship between PTH and the size-adjusted bone mineral content at the femoral trochanter (after adjustment for confounders) was observed, but this relationship was not seen in older Chinese adults (Fig. 3)^(^^87^^)^. This finding was supported by a previous study of elderly men and women from Hong Kong where the bone mineral density at the spine and the hip was not associated with plasma PTH^(^^97^^)^. This suggests that older Chinese adults may have a degree of resistance to the bone resorptive effects of PTH. This was however, not confirmed by a detailed study by Yan *et al.*, as described in the next section.

#### Ethnic differences in renal mineral excretion: China *v.* The UK

There are few studies available directly comparing the differences in renal excretion in these ethnic groups. The comparative results obtained from the 2-h fasting urine samples collected from older Chinese and White-British adults suggested that there were no differences in either the uCa/uCr or the uP/uCr^(^^47^^)^. Yan *et al*. investigated the potential skeletal resistance to PTH in older Chinese adults compared with older White-British adults by conducting a 5-d oral-P loading study to stimulate PTH secretion^(^^47^^)^. No evidence was found for the differences in the skeletal response to PTH and the results did not support the hypothesis of skeletal resistance to PTH in older Chinese adults compared with older British adults. However, a potentially important difference in the renal response to PTH was found, which was greater in the Chinese group, resulting in more rapid P clearance compared with White-British^(^^47^^)^.

### Summary: ethnic differences in Ca and P metabolism

Older people from the Gambia and China have a lower Ca intake and concomitantly higher plasma PTH and 1,25(OH)_2_D concentrations than those from the UK. Yet, unlike in White-British adults, where a high PTH is considered to be a risk factor for osteoporotic fracture, this is not seen in Gambia and China, where the rates of fracture are lower than in the UK. However, while skeletal resistance to PTH is described in African-Americans, this has not been demonstrated in the small studies in older Gambian or Chinese adults.

### Ethnic differences in the diurnal rhythms of Ca and P metabolism

Most of the evidence on the ethnic differences in Ca and P metabolism is derived from studies that have performed investigations under fasting conditions and at standardised times of the day, generally in the early to mid-morning. However, the markers of Ca and P metabolism exhibit a DR, as previously described. By limiting blood or urine sampling to one timepoint along a fluctuating pattern, group differences in the DR or the 24-h mean may go undetected. Little is known about ethnic differences in these DR and their potential influences on the net mineral balance and skeletal health, respectively. Differences in the DR may be anticipated due to known lifestyle differences, such as the total and the pattern of dietary Ca and P intakes, sleep/wake patterns, which are related to known differences in plasma concentrations of PTH and bone turnover markers between ethnic groups.

There is some evidence, albeit limited, to suggest ethnic differences in the DR of PTH and uCa excretion between African-American and White-American women^(^^44^^)^. In the single study investigating these differences, the 24-h mean of plasma PTH concentration was higher in African-Americans and their plasma PTH concentration did not change significantly between day-time and night-time, although uCa excretion (mmol/8 h) was lower at night compared to the day-time. In contrast, White-American women had a significant night-time increase in plasma PTH concentration and a more pronounced nocturnal decrease in uCa (mmol/8 h) than African-Americans. The differences suggest a higher renal conservation of Ca during the day in African-American compared with their White-American counterparts^(^^44^^)^.

This study also demonstrated that ethnic differences in Ca and P metabolism between African-American and White-American women depend on the time of the day. African-Americans had significantly lower uCa and uP (mmol/4 h) compared with White-Americans as measured in the morning and the day-time samples, respectively, but there were no ethnic differences found in uCa or uP (mmol/8 h) in the samples collected during the night^(^^44^^)^.

This highlights the importance of considering the influence of the DR in ethnic comparative research. Therefore, in the interpretation of the differences between ethnic groups, the timing of sample collection needs to be considered. These differences may relate to the timing of the peaks and the nadirs, the amplitude or the 24-h means of the measures of Ca, P and bone metabolism. Also, this may relate to the responses of the bone and kidney to the regulators, which may influence the net effect on Ca and P balances. To address this, we are conducting a comparative study comparing the DR of Ca and P metabolism between older adults from three ethnic groups.

### The investigation of ethnic differences in the diurnal rhythms of Ca and P metabolism

In order to investigate the potential ethnic differences in the DR of Ca and P, we are investigating mineral and bone metabolism in three different ethnic groups: White-British, Gambian and Chinese older adults.

**Fig. 4.**
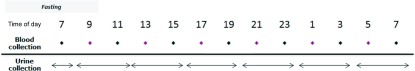
Timings of blood and urine collections.

Each ethnic group is investigated in their habitual environment, thus allowing the participants to follow their normal daily routine. The participants are healthy older men and women (60–75 years). The habitual intake of key nutrients and the timing of their intake are assessed by using methods appropriate to each country, and a sleep/wake pattern questionnaire is administered. Blood samples are collected at 4-h intervals; participants were divided into two groups with a 2-h shift in their sampling times to allow for the analysis of 12 time-points over 24 h (Fig. 4). A 24-h urine collection was carried out, which was divided into seven separate timed collections. The study design allows for the analysis of Ca and P metabolism in the early morning fasting samples, as commonly collected in ethnic comparative research, but also the non-fasting samples at standardised times of the day. Biochemical analyses were performed for the markers of the mineral and the bone metabolism, and their regulators. Data analyses are currently ongoing.

The results of this study will provide information about ethnic differences in Ca, P and bone metabolism, the importance of the sampling time in ethnic comparative research and the potential influence of the DR on the differences in Ca and P metabolism. This will potentially provide mechanistic insights into ethnic differences in mineral and bone metabolism.

## Conclusion

There are striking differences in Ca and P metabolism between ethnic groups, as described by using examples of the differences between the groups within the USA, and between the groups in different countries (The UK, The Gambia and China). These differences in Ca and P metabolism may be associated with the known differences in bone health in older age between these ethnic groups.

Few studies have taken into account the DR of Ca and P metabolism, which are potentially different between groups, as they may be influenced by differences in exogenous factors such as sleep and wake patterns and the absolute amounts and patterns of dietary intake of key nutrients.
